# The relative importance of severity and rarity criteria in health resource allocation: an umbrella review

**DOI:** 10.1017/S0266462324004653

**Published:** 2024-11-14

**Authors:** Mint Chan, Yi Wang, Tanainan Chuanchaiyakul, Kinanti Khansa Chavarina, Wanrudee Isaranuwatchai, Yot Teerawattananon

**Affiliations:** 1 Saw Swee Hock School of Public Health, National University of Singapore, Singapore, Singapore; 2 Health Intervention and Technology Assessment Program (HITAP), Ministry of Public Health, Nonthaburi, Thailand

**Keywords:** resource allocation, technology assessment, biomedical, systematic review

## Abstract

**Objectives:**

The primary objectives of this umbrella review were to (a) quantify the relative importance, of “severity” and “rarity” criteria in health resource allocation; and (b) analyze the contextual factors influencing the relative importance. The secondary objective was to examine how “severity” and “rarity” criteria are defined.

**Methods:**

Searches were carried out in PubMed and Embase to identify eligible systematic reviews. Quality appraisal of systematic reviews was undertaken. From identified systematic reviews, primary studies were extracted and further screened for eligibility. The inclusion of severity and rarity criteria and their respective weights in primary studies were examined. Descriptive and regression analyses were performed.

**Results:**

Twenty-nine systematic reviews were screened, of which nine met the inclusion criteria. Primary studies included in these systematic reviews were retrieved and screened, resulting in forty articles included in the final analysis. Disease severity was more frequently considered *(n* = 29/40) than disease rarity (*n* = 23/40) as an evaluation criterion. Out of all cases where both were included as evaluation criteria, disease severity was assigned higher weights 84 percent of the time (*n* = 21/25).

**Conclusions:**

Our review found consistent evidence that disease severity is more relevant and preferred to rarity as a priority-setting criterion albeit constraints in statistical analysis imposed by limited sample size and data availability. Where funding for rare diseases is concerned, we advocate that decision-makers be explicit in clarifying the significance of disease severity and/or rarity as a value driver behind decisions. Our findings also reinforce the relevance of disease severity as a criterion in priority setting.

## Introduction

Traditionally, health maximization has been the key focus of many health technology assessment (HTA) agencies supporting resource allocation, with the system’s key output being units of health commonly measured by quality-adjusted life years (QALY) (1). However, this approach has been criticized for neglecting nonhealth considerations, such as distribution concerns of who gains and who loses from these decisions and whether those who gain have better or worse off health state as compared to the rest of the population (2;3). This is a limitation as resource allocation solely driven by health maximization could neglect distributive justice and other pertinent social values.

“Disease severity” and “disease rarity” are two pertinent attributes relating to distributive justice. The focus on disease severity as a priority-setting criterion is drawn from several recognized theories relating to distributive justice as well as public preference studies which supports that prioritizing the worst-off is valued by society (4;5). On the other hand, rare diseases have been receiving increasing attention in recent years driven by legislations aimed at improving patient access to treatments (6;7). The relevance of disease severity and rarity as priority-setting criteria is discussed in a number of literatures but attempts to quantitatively determine their relative importance are scarce (4;8;9).

This umbrella review aimed to leverage on the systematic approach and multicriteria nature of existing multi-criteria decision analysis (MCDA) studies to understand how disease severity and rarity are defined, weighted, and traded off in health resource allocation. MCDA is an explicit approach that combines the impact of individual technical and normative value judgment to arrive at a quantitative measure for decision-making, and is increasingly used by a number of HTA agencies including those in Columbia, Germany, Hungary, Italy, Thailand, and the United Kingdom as an alternative or complementary framework for priority setting (10-15).

The primary objectives of this review were to quantify the relative importance of severity and rarity criteria; and analyze the contextual factors influencing their relative importance. The secondary objective was to examine the operating definitions of severity and rarity criteria.

## Methods

An umbrella review, which is a systematic review of existing systematic reviews, was used to provide a broad appraisal of current information available relating to the relative importance of severity and rarity criteria in health resource allocation. This approach was chosen considering the availability of multiple systematic reviews on this topic and the feasibility to consolidate and synthesize quantitative evidence in a time- and resource-efficient manner (16). The protocol of this study is registered on PROSPERO (registration ID CRD42023408265). The reporting of this study was written according to the Preferred Reporting Items for Systematic reviews and Meta-Analyses (PRISMA) 2020 statement (refer to Supplementary Table 7).

### Search strategy

Electronic systematic searches for systematic reviews were carried out on PubMed and Embase databases. The search strategy was guided by the research question and based upon two main concepts: (i) criteria for health resource allocation; and (ii) MCDA as the methodology in priority setting. MCDA was identified as the approach of interest as it allows the identification of studies with a set of assessment criteria beyond the traditional focus on cost utility, and furthermore makes explicit the value contribution of each criterion. This approach allows for the extraction of measurable definitions and relative weights of criteria used in the evaluation. Given that incorporating broader noneconomic considerations into health resource allocation is a relatively recent concept, we restricted our search to the past 10 years – from 1 January 2013 to 31 December 2022. Only articles written in English were included. The detailed search strategy is available in Supplementary Table 1.

### Screening strategy and identification of systematic reviews

One author (CM) screened the title, abstract and full text of systematic reviews identified from search strategies. The inclusion and exclusion criteria were outlined in Supplementary Table 2 and were based on elements in the Population, Phenomena of Interest, Context (PICo) question format (17). Population refers to participants contributing to the weighing of criteria, which could represent a diverse profile including healthcare professionals, policymakers, academics, patient representative, payers, pharmaceutical industry, and general public. We did not apply any restrictions to the participant profile as we were interested in a multistakeholder perspective. Phenomena of interest were identified as criteria used for resource allocation in the health sector, specifically focusing on the consideration and weighing of disease severity and disease rarity. Context refers to value assessments involving MCDA, as its quantitative approach allows for the examination of the performance of individual criteria.

### Screening strategy and identification of primary studies

With eligible systematic reviews identified, primary studies included in each of these systematic reviews were retrieved. There was no restriction in the time period of primary studies included in this review. Primary studies were screened based on the inclusion and exclusion criteria outlined in Supplementary Table 2. Studies that did not fulfill the definition of an MCDA study as stated in Supplementary Table 2 were excluded. In addition, priority setting outside of the context of public health resource allocation and nonhealth fields were excluded. Priority setting in the field of health research was also excluded. Nonprimary research articles such as opinion piece, letters, and editorials were also excluded. Furthermore, studies which are purely methodological hence did not propose a clear list of criteria were also excluded.

Two reviewers (CM and TC) independently screened all primary studies retrieved based on titles and abstracts. Subsequently, full-text articles were screened independently by the same reviewers (CM and TC). For cases of uncertainties or disagreements regarding the inclusion of specific articles, reviewers engaged in discussions until a consensus was achieved.

### Quality assessment

One reviewer (CM) assessed the quality of systematic reviews using AMSTAR 2 tool (18). This tool has sixteen items in total that allow for the assessment of the quality of systematic review that includes either or both the randomized or nonrandomized studies of healthcare interventions. As the AMSTAR 2 is not intended for the generation of a percentage score, a percentage score was calculated according to Fleming et al. (19) to provide an indication of the quality of systematic reviews. Considering that a limited pool of systematic reviews was expected to be identified, no systematic review was excluded in the final analysis based on quality assessment.

### Data extraction

Two data extraction templates were developed, one for systematic reviews and the other for primary studies. After discussing and obtaining consensus from a third reviewer (YT), minor adjustments were made to the data extraction templates. One reviewer (CM) performed data extraction for systematic review. Two independent reviewers (CM and TC) performed data extraction for primary studies.

Data extracted from the systematic review included the time period of analysis, study objective, perspective of analysis, domain of prioritization, geographical region of analysis, and study inclusion and exclusion criteria.

Data extracted from primary studies included whether disease severity and rarity were respectively included as a criterion, their operating definition and weights, the percentile rank of weights of severity and rarity relative to all other criteria considered, year of publication, country of analysis, study objective, funding status, perspective of analysis, domain of prioritization (“sector wide” refers to broad prioritization across health domains/“specific” refers to evaluations on specific disease state or health intervention), level of prioritization (supranational and national/subnational), methods used for preference elicitation, source of criteria, total number of criteria considered, participants involved in criteria setting and weighing respectively. Where information was not applicable or not available, it was reported as “N.A” or “not reported” correspondingly on the data extraction form.

### Data analysis

Descriptive statistics were used to analyze the characteristics of studies in terms of counts and percentage of occurrence. Regression analyses were applied to explore which study characteristics impact the relative importance of severity and rarity criteria. The study characteristics were: (1) year of publication, (2) level of prioritization (supranational and national/subnational), (3) country income level (based on World Bank country classification by income), (4) funding status, (5) domain of prioritization (sector-wide/specific disease state or intervention), (6) perspective of analysis, (7) study objective, (8) source of criteria (proposed by study/adapted from another study), (9) methods used for preference elicitation, (10) participants involved in criteria setting (heterogeneous/homogeneous group), and (11) participants involved in criteria weighing (heterogeneous/homogeneous group) (20).

The following statistical analyses were performed. First, a logistic regression was conducted to understand which study characteristics influence the outcome of whether both severity and rarity criteria were included, or only the severity criterion. As there were only two studies that included rarity, these studies were omitted considering there were too few observations for meaningful analysis. Second, for studies where both criteria were included, the ratio of weights of severity over weights of rarity in each study were computed to generate a score that indicates preference for severity if >1 or rarity if <1. The ratio of weights was taken as the measure for relative importance, as it compares the weight of severity and rarity within the same study thus not subject to bias from heterogeneity across studies. Third, a multivariable regression analysis was conducted to understand which study characteristics influenced the magnitude of the ratio explained above; and sensitivity analyses were conducted to verify the findings from ratio analysis by examining the underlying relationship of severity weights and rarity weights individually with study characteristics to verify if the direction of association was consistent.

Univariate analysis was applied first, followed by multivariable analysis. A forward stepwise selection process was used for model building. Variables were included one by one from the lowest *p*-value to the highest *p*-value based on univariate analysis. The forward stepwise selection ceased when the variable to be added had a *p*-value of more than .1. Considering the relatively small sample size expected, we adopted a more lenient *p*-value of <.1 as the level of statistical significance.

## Results

### Study selection

**Figure 1.**
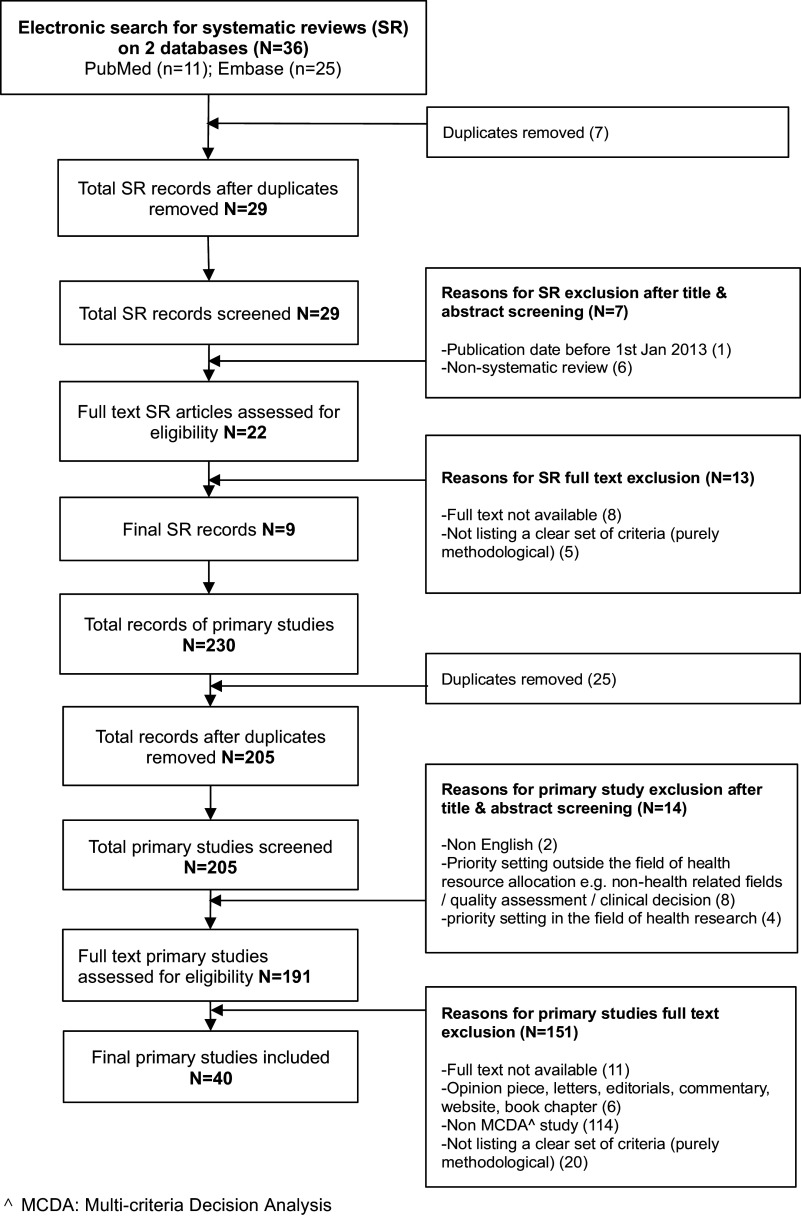
PRISMA chart.

Figure 1 shows the PRISMA flowchart detailing the study selection process. The search from PubMed and Embase returned thirty-six records. After the removal of duplicates, twenty-nine records were screened for title, abstract, and full text review. Based on the inclusion and exclusion criteria, nine systematic reviews were eligible for inclusion. Primary studies included in these systematic reviews were retrieved, which resulted in 230 articles. After excluding duplicates, 205 articles were screened based on title, abstract, and full text. Based on predefined inclusion and exclusion criteria, forty articles were included in the review.

### Quality assessment of systematic reviews

An overview of the included systematic reviews was provided in Supplementary Table 3. Results of quality assessment based on AMSTAR 2 were summarized in Supplementary Table 4. The mean AMSTAR 2 percentage score was 56 percent (standard deviation 18.4), ranging from 26.9 percent to 84.6 percent. As aforementioned, no systematic reviews were excluded based on the quality of assessment in view of a limited pool of studies identified.

### Characteristics of included primary studies

Table 1 summarizes the general characteristics of the included primary studies. Raw data extracted from primary studies were presented in Supplementary Table 5. Among the included studies, fifteen articles were published between 2002 and 2012, and twenty-five articles were published between 2013 and 2019.

**Table 1.** General characteristics of included study

Items	Category	Frequency
Number of included studies		40 (100%)
Year published	2002–2012	15 (37.5%)
	2013–2019	25 (62.5%)
Funding	Received funding	22 (55%)
	Government	9
	Private (for profit)	9
	Private (not for profit)	2
	Mixed funding	2
	No funding	8 (20%)
	Not stated	10 (25%)
Study objective	Ranking of interventions^a^	20 (50%)
	Reimbursement decision-making^b^	16 (40%)
	Normative study^c^	4 (10%)
Domain of prioritization	Sector wide^d^	15 (37.5%)
	Specific^e^	25 (62.5%)
	Pharmaceuticals	7
	Rare disease	7
	Others^f^	11
Level of priority setting	Supranational and national	37 (92.5%)
	Subnational	3 (7.5%)
Country region by income	High income	20 (50%)
	Upper middle income	9 (22.5%)
	Lower middle income	7 (17.5%)
	Low income	0 (0%)
	Not applicable^g^	4 (10%)
Analytical perspective^h^	Reported	12 (30%)
	Policy	5
	Societal	5
	Clinical	1
	Patient	1
	Not specified	28 (70%)
Source of list of criteria	Based on general literature review and stakeholder opinion	22 (55%)
	Adapted from existing studies with modifications	10 (25%)
	Cited list of criteria in existing studies	8 (20%)
Participants involved in criteria setting process	Heterogeneous	18 (45%)
	Homogeneous	14 (35%)
	Not applicable	8 (20%)
	Profile of participants (studies can be counted more than once if they involve more than one category of participants)	
	Policymakers	21
	Academics	16
	Healthcare professionals	13
	Patient representatives	8
	General public	5
	Pharmaceutical industry	3
Participants involved in criteria weighing process	Heterogeneous	22 (55%)
	Homogeneous	11 (27.5%)
	Not applicable	7 (17.5%)
	Profile of participants (studies can be counted more than once if they involve more than one category of participants)	
	Policymakers	24
	Healthcare professionals	18
	Patient representatives	12
	Academics	9
	General public	6
	Pharmaceutical industry	3
	Payers	3
Preference elicitation technique^i^	Discrete choice experiment	11 (27.5%)
	Non-hierarchical point allocation	9 (22.5%)
	Hierarchical point allocation	7 (17.5%)
	Others	6 (15%)
	Not applicable^j^	7 (17.5%)
Severity criterion considered in priority setting	Yes	29 (72.5%)
	Definition provided	25
	Proposed by study	10
	As defined by EVIDEM framework^k^	9
	Cited existing studies	6
	No definition provided	4
	No	11 (27.5%)
Rarity criterion considered in priority setting	Yes	23 (57.5%)
	Definition provided	17
	Proposed by study	8
	As defined by EVIDEM framework	6
	Cited existing studies	3
	No definition provided	6
	No	17 (42.5%)

a Rank ordering of a list of interventions based on performance established from a predefined set of criteria.

b Reimbursement decision-making based on performance established from a predefined set of criteria.

c Normative study refers to studies with a main objective to understand criteria, instead of comparing interventions.

d Sector wide refers to establishing a set of criteria for priority setting irrespective of any healthcare intervention(s)/disease state.

e Specific domain of prioritization refers to establishing a set of criteria for priority setting in the context of specific healthcare intervention(s)/disease state.

f Counted under “others” for specific priority setting context that has less than 3 counts.

g Studies which involved countries belonging to different income categories.

h Analytical perspective refers to the perspective of the study from which the evaluation is carried out, for instance the evaluation could be conducted from a policy, healthcare or societal perspective.

i Preference elicitation technique refers to systematic methods that elicit and quantify preference of stakeholders with respect to different health interventions or outcomes.

j Not applicable if study does not involve preference elicitation from stakeholders.

k EVIDEM: Evidence and Value: Impact on Decision Making framework.

In terms of the origin of criteria used, approximately half (*n* = 22, 55 percent) of included studies developed their own list of priority-setting criteria based on general literature review and stakeholder opinion; 25 percent (*n* = 10) adopted a set of criteria from specific studies, whereas remaining 20 percent (*n* = 8) adopted list of criteria from existing studies. Among the thirty-two studies that developed/adapted their own list of criteria, the majority (*n* = 22, 69 percent) involved more than one group of stakeholders in the criteria setting. Policymakers were involved in the criteria setting in approximately half of the studies (*n* = 21, 52.5 percent), followed by academics (*n* = 16, 40 percent) and healthcare professionals (*n* = 13, 32.5 percent).

### Relative importance of severity and rarity criteria

The median percentile rank of disease severity weights, relative to all other criteria within the same study, was 72 percent (standard deviation 27.8 percent), whereas the median percentile rank of disease rarity was 34 percent (standard deviation 27.4 percent). This indicates that disease severity was strongly prioritized, with 72 percent of other criteria receiving lower weights. Conversely, although disease rarity had a lower median percentile rank of 34 percent, it is still of considerable importance, as it was given higher weight than one-third of the other criteria assessed.

Disease severity was more frequently considered (*n* = 29/40, 72.5 percent) than disease rarity (*n* = 23/40, 57.5 percent) as an evaluation criterion in the included studies. Eighteen studies have considered both disease severity and rarity as evaluation criteria. Out of these eighteen studies, four studies performed subgroup analysis, which was based on either stakeholder group or country. Thus, with some studies having more than one set of output on weights of severity and rarity, these gave rise to a total of 25 pairs of severity–rarity weights from these eighteen studies.

**Figure 2.**
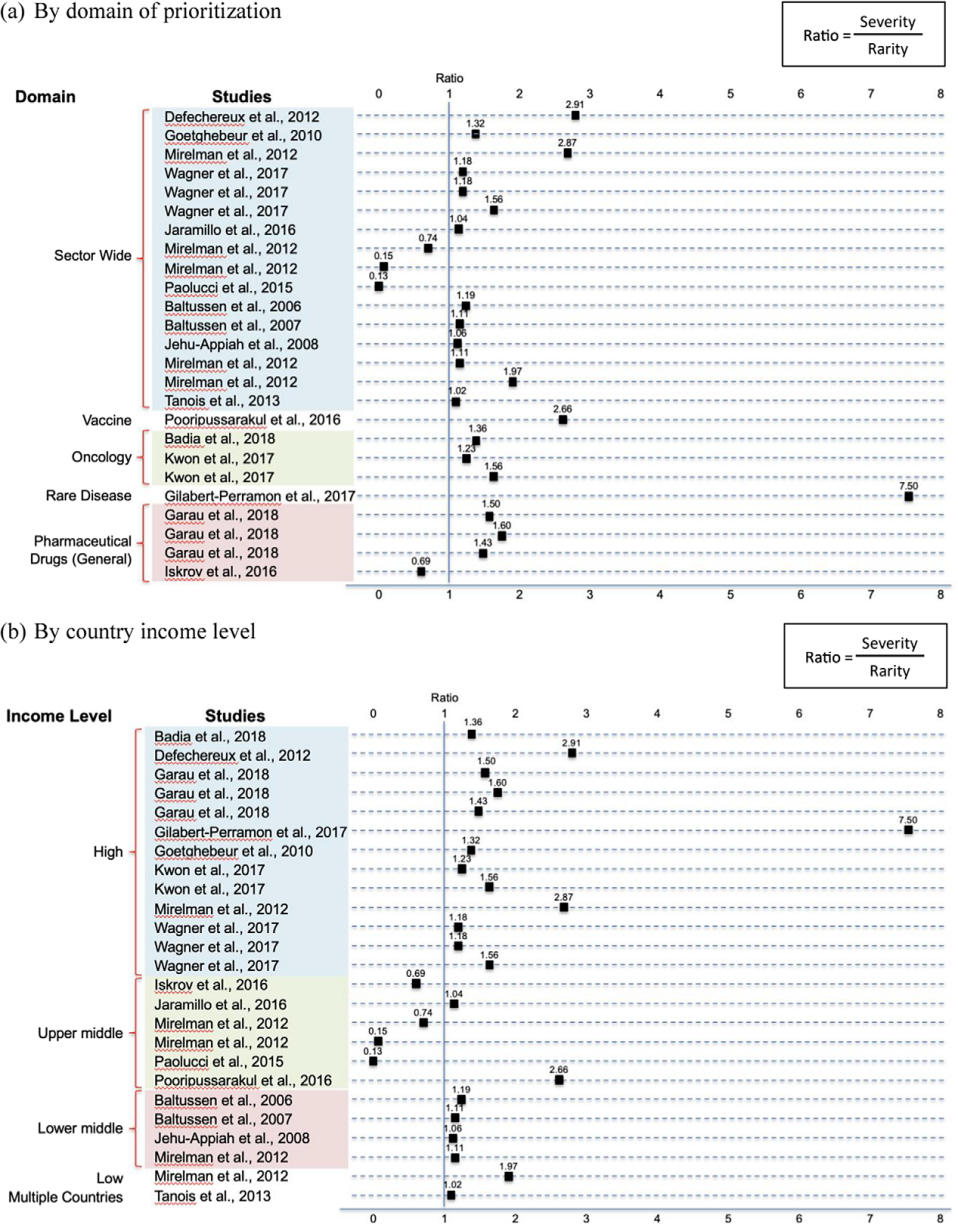
Ratio of weights of disease severity over disease rarity.

Out of the twenty-five cases that considered both as evaluation criteria, disease severity was assigned higher weights than rarity in twenty-one cases. The ratio of severity over rarity (i.e., 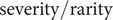 ) was computed as an indicator for preference, with <1 indicating preference for rarity and > 1 indicating preference for severity. Twenty-one cases had a ratio of >1, implying a higher preference for the severity attribute over rarity in most cases. Notably, the study by Gilabert-Perramon et al. (21) on evaluation criteria for orphan drugs represented an outlier with a ratio of 7.50. In this study, severity was given the highest weight (15.0) and rarity was given the lowest weight (2.0) amongst fifteen other criteria based on the hierarchical point allocation technique, implying a strong relative preference for the severity criterion. A graphical representation of the ratio of weight of severity over rarity of these twenty-five cases is shown in Figure 2.

### Factors influencing the relative importance of severity and rarity criteria

Results of all regression analyses conducted were compiled and presented in Supplementary Table 6.

When univariate analysis was run to analyze the association between the magnitude of ratio and potential predictor variables, only income of country was found to be statistically significant. Thus, model building for multivariate regression analysis was not conducted. By interpreting the regression coefficient, high-income countries had a ratio value that is higher than non-high-income countries by 1.02 (*p* = .069), the association was statistically significant taking *p* < .1 as the significance level.

When univariate logistic regression analysis was applied to examine which study characteristics affected the outcome of whether both criterion or only severity was included, no variables were found to be significant. Only domain of prioritization was found to be near statistical significance (*p* = .106); hence, model building for multivariate analysis was not carried out. Based on the regression results, the odds of considering severity only rather than both criteria were 6.42 times higher for specific domain than sector wide. This implies that when evaluations were conducted on specific healthcare intervention(s) or disease state(s) as compared to a broad sector-wide prioritization across health domains, disease severity was found to be a more relevant criterion than rarity with near statistical significance (*p* = .106).

### Sensitivity analysis

Separate univariate analysis with weights of severity and rarity respectively found more significant predictor variables compared to when its ratio within the same study was taken for analysis. Apart from “income of country” which was a significant predictor variable for the ratio of severity over rarity, the separate univariate analysis found “source of criteria” to have a significant effect on both the weights of severity and rarity separately. In addition, “methods used for preference elicitation” were also found to have a significant effect but only on the weights of severity and not for rarity. Both were not significant predictor variables for the ratio of severity over rarity.

Sensitivity analysis revealed the same direction of relationship which was consistent with the observation that the ratio of weights of severity over rarity was higher in high-income countries compared to non-high-income countries. In the univariable regression analysis examining the relationship between the weight of severity and country income, a −0.505 (*p* = .0619) coefficient implied that the mean severity weight was lower by 0.505 in non-high-income countries compared to high-income countries. On the other hand, in the univariable regression analysis examining the relationship between the weight of rarity and country income, a +0.331 (*p* = .0752) coefficient implied that the mean rarity weight was higher by 0.331 in non-high-income countries compared to high-income countries. However, both coefficients were not statistically significant in the multivariable regression analysis.

### Definition of severity criterion used

Out of the twenty-nine studies which considered severity as a criterion, twenty-four reported a definition for severity. Five studies did not state a definition. It was unclear if these studies did not employ an explicit definition, or that the definition was not reported.

Variation in definitions used was observed across studies. Disease severity was observed to be defined in terms of quality of life, length of life, or both. A majority of studies (*n* = 22/25, 88 percent) consider both quality and length of life in its definition. Most studies which had a descriptive definition referred to the Evidence and Value: Impact on Decision Making (EVIDEM) framework, which generically defined severity as the health condition “with respect to mortality, disability, impact on quality of life, clinical course (i.e., acuteness, clinical stages)” (22). Among studies that adopted a quantifiable definition of severity, the most common approach was to quantify based on the expected/remaining QALY of patients living with a particular health condition. In terms of the specific QALY threshold set, four studies applied a QALY threshold of ≤2, whereas 2 studies applied a QALY threshold of ≤5 to indicate severe disease.

### Definition of rarity criterion used

In contrast with the severity criterion, quantifiable definitions were more commonly observed (*n* = 10) for the rarity criterion. Seven studies used a descriptive definition for rarity. Rarity is commonly defined either in terms of the proportion of the population affected by the condition or based on the absolute number of people affected per year. Four of studies applied a scale that describes different extents of rarity rather than a single cut-off threshold, but none defined an interpretation to the cut-offs, for instance, “rare” or “ultra-rare” and so forth. Variation existed in the proportion of population defined as rare, for instance, in the context of orphan drugs, Schey C et al. (23) defined three levels of rarity, with lowest level being 1:20,000 and highest level being <1:200,000; whereas Kolasa et al. (24) defined the lowest level as >1:10,000 and highest level as <1:20,000.

## Discussion

This review examined how the severity and rarity criteria were defined and valued in different contexts of health resource allocation. To our knowledge, no similar publication attempted to quantify the trade-off between severity and rarity and to evaluate factors influencing their relative importance. A few studies examined the trade-off within different levels of severity but not relative to rarity (24-26).

### Relative importance of severity and rarity criteria

It was consistently observed from our review that disease severity was more relevant than rarity as a priority-setting criterion. This finding was concluded considering: (1) more studies included the severity criterion than rarity; (2) multivariable logistic regression showed preliminary findings that severity alone is six times more likely to be considered than rarity when a specific disease state(s)/ intervention(s) was examined; and (3) in studies where both criteria were considered, severity was also found to be more preferred to rarity as weights ratio favored severity in all except four out of twenty-five studies (84 percent).

The association that severity was more relevant than rarity when specific disease states were evaluated appears to be driven by several included studies on rare diseases that did not necessarily consider the rarity criterion. Despite the prevalence of rare diseases could vary considerably, ranging from less than one in a million to a few hundred per million individuals, only the severity criterion was considered with no special merit given to rare diseases with lower prevalence showing that severity is more of a value driver than rarity for rare diseases (27).

On the other hand, for studies on rare diseases that did include both disease severity and rarity as criteria, overall lower weights were assigned to disease rarity by stakeholders as compared to that for disease severity. The high outlier ratio score drawn from Gilabert-Perramon et al. (21) on orphan drugs similarly supported the observation that disease severity appears to be the main value driver in the priority-setting for rare diseases. Multiple preference-based studies conducted among the general public in the United States, Canada, and Norway, also found that rarity, when considered in isolation, was not seen as a healthcare priority by the society (28-30).

### Factors influencing the relative importance of severity and rarity criteria

Healthcare priority including the preference for severity or rarity could depend on stage of economic development and resources available to a country (31;32). When regression analysis was run to examine variables that affected the ratio of severity over rarity, it was observed that a high country income was significantly associated with a 1.02 point higher ratio, as compared to non-high-income category (upper middle, lower middle, and low income). Differences in relative weights assigned to severity and rarity criteria could potentially be due to the disparity in the capacity to handle severe diseases between high-income countries and non-high-income countries (upper middle, lower middle, and low income countries).

It is noteworthy that the inclusion or exclusion of criteria could depend on the scope of evaluation. In certain cases, criteria could be implicitly captured in the selection of disease states for evaluations, thus not included as evaluation criteria, such as in Angelis et al. (33) where neither disease severity nor rarity/prevalence were specified as a criterion as it is implicit in the selection of metastatic colorectal cancer as the topic for evaluation. In other cases, certain evaluation criteria could be less relevant or irrelevant depending on the scope of evaluation, such as in the study by Roldan et al. (34) where “rarity” was not considered as the resource allocation exercise is in the context of a small setting of hospital formulary listing.

### Definition of disease severity and rarity

Most studies included in our review defined disease severity in terms of both the quality and length of life. This corroborates with National Institute for Health and Care Excellence (NICE)’s new “severity modifier” introduced in 2022 that considers both dimensions as compared to the previous narrower “end-of-life” definition which only takes into account the length of life (35;36).

We observed that the severity definition in our review tends to be more simplistic and straightforward, owing to the nature of MCDA preference studies, which captures different concepts as distinct attributes to analyze the impact of each attribute. Operationalized definitions by public health agencies, including NICE and those in Norway and the Netherlands, tend to be more multidimensional, which could include concepts of fair innings and unmet need by quantifying QALY shortfall or taking into account the current standard of care in evaluating disease severity (35;37;38).

There is no universal consensus on the definition of rare disease at present (6). The threshold used to define rarity remains highly varied across jurisdictions, which was similarly observed in our review. All definitions observed in our review were purely prevalence-based due to the nature of MCDA studies, unlike operationalized definitions which could include qualifiers pertaining to disease severity or unmet need (6).

### Policy implications and recommendations

Rare diseases are often also severe diseases (39;40). Our findings argue against prioritizing based on rarity attribute alone, but should not be confused with prioritizing rare disease itself. In light of the increasing number of programs to improve access to rare disease treatments, our review could be helpful to inform the development of an evaluation framework for rare disease treatment (7;41). Additionally, the finding that severity is as or more relevant and preferred to rarity as a priority setting criteria advocates for decision-makers to make explicit the primary value driver behind funding decisions for rare diseases.

Incorporating broader societal values into resource allocation decisions and furthermore demonstrating transparency in the process would enhance public trust and strengthen the legitimacy of HTA as decision-making tool (42). The framework developed in 2023 by Charlton et al., (43) a cross-disciplinary collaboration among twenty-four experts in healthcare priority-setting, could be a useful starting point to facilitate discussions on articulating normative values into case-based judgments. This framework addresses the conceptual ambiguity in normative reasoning, which is often the pain point in incorporating normative values into value assessment, by clarifying different types of normative commitments. It provides a structured framework which enables coherent and transparent reasoning that is capable of withstanding ethical examination.

### Strengths and Limitations

A strength of this study was the consolidation of weights of severity and rarity from all eligible preference-based studies, which provides valuable quantitative evidence on the relative importance of severity and rarity that is scarce in the literature. However, findings were preliminary due to constraints in statistical analysis imposed by small sample size and data availability in primary studies. Heterogeneity in the definition of severity and rarity and methods used to elicit preference could also influence the robustness of our findings, despite attempts to control for these variables in regression analysis. Another limitation was that only peer reviewed literature was included in our analysis; and therefore, the current study excluded grey literature such as government documents, technical reports, conference proceedings which could contain valuable data; an area where future research could consider.

## Conclusion

There is preliminary evidence found from this review that disease severity could be more relevant than and preferred to rarity as a priority-setting criterion. In the context of rare disease funding, decision-makers could consider being more explicit in clarifying whether and to what extent a decision is primarily based on considerations for disease severity or rarity or other attributes.

## Supporting information

Chan et al. supplementary material Chan et al. supplementary material

## Supplementary material

The supplementary material for this article can be found at http://doi.org/10.1017/S0266462324004653.
