# Supplementary material for: The relative importance of severity and rarity criteria in health resource allocation: an umbrella review
Source: Int J Technol Assess Health Care. 2024 Nov 14;40(1):e54. doi: 10.1017/S0266462324004653 (PMC11579674; doi:10.1017/S0266462324004653)
Supplement: Chan et al. supplementary material [file S0266462324004653sup001.zip › Supplementary file 1_CHAN.docx]

**Supplementary Table 1 – Search Strategy**

| **Search No.** | **Search area** | **Search** | **Date of search** | **Number of hits** |
| --- | --- | --- | --- | --- |
| **Pubmed** (Medline) |  |  |  |  |
| #1 | Resource allocation | “reimbursement*” [Title/Abstract] OR “coverage”[Title/Abstract] OR “subsid*”[Title/Abstract] OR “inance*”[Title/Abstract] OR “resource allocation*”[Title/Abstract] OR “allocation*”[Title/Abstract] OR “distribution*” [Title/Abstract] OR priorit*[Title/Abstract] OR “priority setting”[Title/Abstract] OR “rationing”[Title/Abstract] OR “health technology assessment*”[Title/Abstract] OR “HTA” [Title/Abstract] OR “technology assessment, biomedical”[MeSH Terms] OR “biomedical technology assessment*”[Title/Abstract] | 31 Dec 2022 | 21,835 |
| #2 | Decision making criteria | “Criteria*”[Title/Abstract] OR “Scoring”[Title/Abstract] OR “Value*”[Title/Abstract] OR “weighting”[Title/Abstract] | 31 Dec 2022 | 3,039,222 |
| #3 | Health equity | “Equity”[Title/Abstract] OR “Health equity”[Title/Abstract] OR “Health equity”[MeSH Terms] OR “Fairness”[Title/Abstract] OR “social justice”[MeSH Terms] OR “social justice”[Title/Abstract] OR “severity of illness index” [MeSH Terms] OR “disease severity”[Title/Abstract] OR “severity of illness index”[Title/Abstract] OR “rare”[Title/Abstract] OR “rarity”[Title/Abstract] OR “minority health”[MeSH Terms] OR “minority health”[Title/Abstract] OR “Health Disparity, Minority and Vulnerable Populations”[MeSH Terms] OR [“Health Disparity, Minority and Vulnerable Populations”[Title/Abstract] | 31 Dec 2022 | 1,211,392 |
| #4 | Healthcare | “health” [Title/Abstract] OR “healthcare”[Title/Abstract] OR “medicine*”[Title/Abstract] OR “medication*” [Title/Abstract] OR “disease*” [Title/Abstract] OR “pharmaceutical*” [Title/Abstract] OR “drug*” [Title/Abstract] OR “therapy” [Title/Abstract] OR “therapies” [Title/Abstract] OR “treatment*” [Title/Abstract] OR “medical device*” [Title/Abstract] | 31 Dec 2022 | 12,484,078 |
| #5 | MCDA | “multicriteria”[Title/Abstract] OR “Multi-criteria”[Title/Abstract] OR “multiple criteria”[Title/Abstract] OR “Multi-criteria decision analysis”[Title/Abstract] OR MCDA[Title/Abstract] OR MCDM[Title/Abstract] OR “multi-attribute decision analysis”[Title/Abstract] | 31 Dec 2022 | 4,583 |
| #6 | Combined search | #1 AND #2 AND #3 AND #4 AND #5 | 31 Dec 2022 | 101 |
| #7 | Combined search | Filters: systematic review | 31 Dec 2022 | **11** |
| #8 | Combined search | Filters: English, systematic review Publication date: 01/01/2013 – date of search | 31 Dec 2022 | **10** |

| **Search No.** | **Search area** | **Search** | **Date of search** | **Number of hits** |
| --- | --- | --- | --- | --- |
| **Embase** |  |  |  |  |
| #1 | Resource allocation | ‘reimbursement*’:ti,ab OR ‘reimbursement’/exp OR coverage:ti,ab OR ‘subsid*’:ti,ab OR ‘finance’/exp OR ‘financ*’:ti,ab OR ‘resource allocation’:ti,ab OR ‘resource allocation’/exp OR ‘allocation*’:ti,ab OR ‘distribution*’:ti,ab OR ‘priorit*’:ti,ab OR ‘priority setting’:ti,ab OR ‘rationing’:ti,ab OR ‘HTA’:ti,ab OR ‘heath technology assessment’:ti,ab OR ‘biomedical technology assessment’/exp OR ‘biomedical technology assessment’:ti,ab | 31 Dec 2022 | 2,980,393 [^42^](#_ENREF_42) |
| #2 | Decision making criteria | ‘Criteria*’:ti,ab OR Scoring:ti,ab OR ‘Value*’:ti,ab OR ‘Equity weighting’:ti,ab | 31 Dec 2022 | 4,083,305 |
| #3 | Health equity | Equity:ti,ab OR ‘Health equity’:ti,ab OR ‘Health equity’/exp OR Fairness:ti,ab OR ‘social justice’/exp OR ‘social justice’:ti,ab OR ‘severity of illness index’/exp OR ‘disease severity’/exp OR ‘disease severity’:ti,ab OR ‘disease severity assessment’/exp OR ‘disease severity assessment’:ti,ab OR ‘severity of illness index’:ti,ab OR rare:ti,ab OR rarity:ti,ab OR ‘minority health’/exp OR ‘minority health’:ti,ab OR ‘Vulnerable population’/exp OR ‘Vulnerable population’:ti,ab | 31 Dec 2022 | 3,267,197 |
| #4 | Healthcare | ‘health’/mj OR health:ti,ab OR ‘healthcare’/mj OR healthcare:ti,ab OR ‘medicine’/exp OR medicine:ti,ab OR ‘medication’/exp OR medication:ti,ab OR ‘disease’/exp OR disease:ti,ab OR pharmaceutical:ti,ab OR ‘drug’/exp OR drug:ti,ab OR ‘therapy’/exp OR therapy:ti,ab OR therapies:ti,ab OR ‘treatment’/exp OR treatment:ti,ab OR ‘health technolog*’:ti,ab OR ‘health intervention*’:ti,ab OR ‘medical device’/exp OR ‘medical device’:ti,ab | 31 Dec 2022 | 32,972,954 |
| #5 | MCDA | multicriteria:ti,ab OR ‘multiple criteria’:ti,ab OR mcda:ti,ab OR mcdm:ti,ab OR ‘multi-attribute decision analysis’:ti,ab OR ‘multicriteria decision analysis’/exp OR ‘multicriteria decision analysis’:ti,ab | 31 Dec 2022 | 6,854 |
| #6 | Combined search | #1 AND #2 AND #3 AND #4 AND #5 | 31 Dec 2022 | 228 |
| #7 | Combined search | Filters: systematic review | 31 Dec 2022 | 21 |
| #8 | Combined search | Filters: English, systematic review Publication date: 01/01/2013 – date of search | 31 Dec 2022 | **24** |
