# Supplementary material for: The relative importance of severity and rarity criteria in health resource allocation: an umbrella review
Source: Int J Technol Assess Health Care. 2024 Nov 14;40(1):e54. doi: 10.1017/S0266462324004653 (PMC11579674; doi:10.1017/S0266462324004653)
Supplement: Chan et al. supplementary material [file S0266462324004653sup001.zip › Supplementary file 2_CHAN.docx]

**Supplementary Table 2 – Literature selection process**

| The literature screening was conducted in two steps: |
| --- |
| 1. Screening of systematic reviews. |
| 1. Title and abstract screening of identified records by two independent researchers. |
| The following exclusion criteria were hierarchically applied during title-abstract screening: |
| 1. *Publication date outside of 1 Jan 2013 – 31 Dec 2022* |
| 1. *Non-english language* |
| 1. *Non-systematic review on criteria for health resource allocation* |
| Articles not fitting any of these exclusion criteria were eligible for full text screening. |
| 1. Full text screening of potentially relevant systematic reviews by two independent researchers. |
| The following exclusion criteria were hierarchically applied: |
| 1. *Full text not found* |
| 1. *MCDA in fields other than human health care*; |
| 1. *Not listing a clear set of criteria (purely methodological)* |
| Articles not fitting any of these exclusion criteria were eligible. |
|  |
| 1. Screening of primary studies. |
| Based on systematic reviews identified, primary studies included in each systematic reviews were retrieved for screening. |
| 1. Title and abstract screening of all retrieved primary studies by two independent researchers. |
| The following exclusion criteria were hierarchically applied during title-abstract screening: |
| 1. *Non-english language* |
| 1. *Priority setting outside the field of health resource allocation e.g. non-health related fields / quality assessment / clinical decision* |
| 1. *Priority setting in the field of health research* |
| Articles not fitting any of these exclusion criteria were eligible for full text screening. |
| 1. Full text screening of potentially relevant primary studies by two independent researchers. |
| The following exclusion criteria were hierarchically applied: |
| 1. *Full text not found* |
| 1. *Not original research e.g. opinion piece, letters, editorials, commentary, website, book chapter* |
| 1. *Non MCDA study. MCDA study is defined in this review as study which used a set of methods to aid in health resource allocation decision making, where decisions are based on more than one criterion. It uses available techniques ranging from simple summation to mathematical models to assign weights* *to predefined criteria to allow for a rational and transparent approach that makes explicit the impact of criteria on the outcome of health resource allocation decision making.* |
| 1. *Not listing a clear set of criteria (purely methodological)* |
| Articles not fitting any of these exclusion criteria were eligible. |
