# Supplementary material for: The relative importance of severity and rarity criteria in health resource allocation: an umbrella review
Source: Int J Technol Assess Health Care. 2024 Nov 14;40(1):e54. doi: 10.1017/S0266462324004653 (PMC11579674; doi:10.1017/S0266462324004653)
Supplement: Chan et al. supplementary material [file S0266462324004653sup001.zip › Supplementary file 3_CHAN.docx]

|  | **Supplementary Table 3 – Overview of Included Systematic Reviews (N=9)** | | | | | | | | |
| --- | --- | --- | --- | --- | --- | --- | --- | --- | --- |
| **Index** | S1 | S2 | S3 | S4 | S5 | S6 | S7 | S8 | S9 |
| **Title** | Systematic review on the evaluation criteria of orphan medicines in Central and Eastern European countries | The health systems' priority setting criteria for selecting health technologies: A systematic review of the current evidence | Criteria Used for Priority-Setting for Public Health Resource Allocation in Low- and Middle- Income Countries: A Systematic Review | Multi-Criteria Decision Analysis (MCDA) Models in Health Technology Assessment of Orphan Drugs—a Systematic Literature Review. Next Steps in Methodology Development? | What Is Value in Health and Healthcare? A Systematic Literature Review of Value Assessment Frameworks | Criteria and Scoring Functions Used in Multi‐criteria Decision Analysis and Value Frameworks for the Assessment of Rare Disease Therapies: A Systematic Literature Review | Using Economic Evidence to set Healthcare Priorities in Low-income and Middle-income Countries: A Systematic Review of Methodological Frameworks. | Amplifying Each Patient’s Voice: A Systematic Review of Multi-criteria Decision Analyses Involving Patients | Value Assessment of Oncology Drugs Using a Weighted Criterion-Based Approach |
| **Authors** | Zelei et al. | M. Mobinizadeh, et al. | G. Kaur et al. | Baran-Kooiker et al. | Zhang et al. | T. Zelei et al. | Wiseman et al. | K. Marsh et al. | Ezeife et al |
| **Year** | 2016 | 2016 | 2019 | 2018 | 2022 | 2021 | 2016 | 2017 | 2020 |
| **Time period** | Jan 2000 - Apr 2015 | Up to Mar 2015 | 2000-2016 | Up to 1st January 2018 | 2008 - 2019 | 2013-2019 | 2004-2014 | Up to Jun 2014 | Up to Aug 2018 |
| **Perspective of analysis** | Payer | N/A | Policy | N/A | N/A | N/A | N/A | Patient | N/A |
| **Priority setting context** | Orphan drugs | Health technologies | Healthcare interventions | Orphan drugs | Healthcare interventions | Rare disease | Healthcare interventions | Healthcare interventions | Oncology drugs |
| **Objective** | To list all potentially relevant value drivers in the reimbursement process of orphan drugs | To identify the most appropriate health technologies priority setting criteria  and their applications | To identify criteria being used for priority setting for resource allocation decisions in low- and middle-income countries (LMICs) | To provide an overview of the current state of knowledge and latest developments in the field of MCDA | To investigate how value is defined and measured in existing value assessment frameworks (VAFs) in healthcare | To investigate the criteria and scoring functions applied in value frameworks and MCDA tools | To synthesize and appraise the literature on methodological frameworks – which incorporate economic evaluation evidence – for the purpose of setting healthcare priorities in LMICs | To report on existing MCDAs involving patients to support the future use of MCDA to capture the patient voice | To establish the drug assessment framework (DAF), with key criteria identified and weighted by multistakeholder group. |
| **Level of priority setting** | Central and Eastern European | N/A | LMICs | N/A | N/A | N/A | LMICs | N/A | N/A |
| **Main Inclusion Criteria** | - | (1) Papers and academic theses which included specified qualitative and quantitative criteria;  (2) Fall in line with priority setting of health technologies. | Any study that referred to relevant criteria or process for decision making | MCDA for OMP/rare diseases on the subject of: (1) model creation and adjustment, (2) identification and definition of model criteria, weight elicitation, model validation  (3) impact of MCDA application on decision making. | (1) The study output is a VAF  (2) The intended use of the framework is to support HTA and inform decision making | (1) Contained explicit scoring functions for the included criteria and were orphan drug specific  (2) Articles which were considered ‘referenced’ general framework | (1) Reported on at least one ‘low’-income or ‘lower-middle’-income country as defined by the World Bank   (2) Reported on priority-setting frameworks or approaches | (1) Reported the application of MCDA to assess healthcare interventions  (2) MCDAs that involved patients as a source of weights | Not reported |
| **Main exclusion criteria** | (1) Clinical papers without reference to HTA  (2) General paper that describe environment for ODs without reference to HTA | (1) Nonconformity to priority setting framework for health technologies  (2) lack of specified qualitative and quantitative criteria | (1) Studies on defining research priority setting  (2) Opinion pieces and editorials  (3) Studies/ reports on high-income countries | (1) Did not address MCDA and OMPs in-depth or as the main subject  (2) Subject of MCDA outside the field of HTA and/or reimbursement for OMPs | 1) Developing frameworks for other purposes  (2) Identifying or measuring specific value attributes but not for framework development  (3) Evaluating or testing an existing value framework;  (4) Protocols, clinical practice guidelines, comments, or opinions about VAFs. | (1) Not related to MCDA/value frameworks  (2) MCDA/value frameworks in fields other than human health care;  (3) Scope other than pharmaceuticals  (4) Not listing a clear set of criteria  (5) MCDA tool/value framework supporting patient-level or shared decision making or clinical /hospital level decision making;  (6) No explicit measurement method/no explicit scoring function. | - | Did not apply MCDA | Not reported |
