# Supplementary material for: The relative importance of severity and rarity criteria in health resource allocation: an umbrella review
Source: Int J Technol Assess Health Care. 2024 Nov 14;40(1):e54. doi: 10.1017/S0266462324004653 (PMC11579674; doi:10.1017/S0266462324004653)
Supplement: Chan et al. supplementary material [file S0266462324004653sup001.zip › Supplementary file 4_CHAN.docx]

|  | **Supplementary Table 4 - Quality of Reporting based on AMSTAR 2** | | | | | | | | | |
| --- | --- | --- | --- | --- | --- | --- | --- | --- | --- | --- |
|  | Studies (n=9) | 1 | 2 | 3 | 4 | 5 | 6 | 7 | 8 | 9 |
| 1 | Did the research questions and inclusion criteria for the review include the components of PICO? | Yes | Yes | Yes | Yes | Yes | Yes | Yes | Yes | Yes |
| 2 | Did the report of the review contain an explicit statement that the review methods were established prior to the conduct of the review and did the report justify any significant deviations from the protocol? | No | No | Yes | Partial yes | Partial yes | Partial yes | No | Partial yes | Partial Yes |
| 3 | Did the review authors explain their selection of the study designs for inclusion in the review? | Yes | Yes | Yes | Yes | Yes | Yes | Yes | Yes | Yes |
| 4 | Did the review authors use a comprehensive literature search strategy? | Yes | Partial yes | Partial yes | Partial yes | Partial yes | Yes | Partial yes | Partial yes | Partial yes |
| 5 | Did the review authors perform study selection in duplicate? | Yes | No | Yes | No | Yes | Yes | No | Yes | No |
| 6 | Did the review authors perform data extraction in duplicate? | No | No | No | No | Yes | No | No | No | No |
| 7 | Did the review authors provide a list of excluded studies and justify the exclusions? | No | No | No | No | No | No | No | No | No |
| 8 | Did the review authors describe the included studies in adequate detail? | Yes | Yes | Yes | Yes | Yes | Yes | Yes | Yes | Partial yes |
| 9 | Did the review authors use a satisfactory technique for assessing the risk of bias (RoB) in individual studies that were included in the review? | No | No | Yes | Yes | Yes | Partial yes | Partial yes | Partial yes | Partial yes |
| 10 | Did the review authors report on the sources of funding for the studies included in the review? | Yes | No | No | Yes | Yes | No | No | No | No |
| 11 | If meta-analysis was performed did the review authors use appropriate methods for statistical combination of results? | N/A | N/A | N/A | N/A | N/A | N/A | N/A | N/A | N/A |
| 12 | If meta-analysis was performed, did the review authors assess the potential impact of RoB in individual studies on the results of the meta-analysis or other evidence synthesis? | N/A | N/A | N/A | N/A | N/A | N/A | N/A | N/A | N/A |
| 13 | Did the review authors account for RoB in individual studies when interpreting/discussing the results of the review? | No | No | No | Yes | Yes | Yes | Yes | No | No |
| 14 | Did the review authors provide a satisfactory explanation for, and discussion of, any heterogeneity observed in the results of the review? | Yes | No | No | Yes | Yes | Yes | Yes | No | No |
| 15 | If they performed quantitative synthesis did the review authors carry out an adequate investigation of publication bias (small study bias) and discuss its likely impact on the results of the review? | N/A | N/A | N/A | N/A | N/A | N/A | N/A | N/A | N/A |
| 16 | Did the review authors report any potential sources of conflict of interest, including any funding they received for conducting the review? | Yes | No | Yes | Yes | Yes | Yes | Yes | Yes | Yes |
|  | % score | 61.5 | 26.9 | 57.6 | 69.2 | 84.6 | 69.2 | 53.8 | 50.0 | 30.8 |
| ^1^Zelei et al. ^2^M.Mobinizadeh et al. ^3^G.Kaur et al. ^4^Baran-Kooiker et al. ^5^Zhang et al. ^6^T.Zelei et al. ^7^Wiseman et al. ^8^K.Marsh et al. ^9^Ezeife et al. | | | | | | | | | | |
