# Supplementary material for: The relative importance of severity and rarity criteria in health resource allocation: an umbrella review
Source: Int J Technol Assess Health Care. 2024 Nov 14;40(1):e54. doi: 10.1017/S0266462324004653 (PMC11579674; doi:10.1017/S0266462324004653)
Supplement: Chan et al. supplementary material [file S0266462324004653sup001.zip › Supplementary file 5_CHAN.docx]

|  | **Supplementary Table 5 – Overview of included primary studies (N=40)** | | | | | | | | | | | | | | |
| --- | --- | --- | --- | --- | --- | --- | --- | --- | --- | --- | --- | --- | --- | --- | --- |
| **S/N** | **Title** | **Year** | **Country** | **Domain** | **Perspective of analysis** | **Preference elicitation technique^#^** | **Total number of criteria considered** | **Severity** | | |  | **Rarity** | | | |
|  |  |  |  |  |  |  |  | **Included**  **(Y/N)** | **Definition** | **Weights** | **Percentile Rank** | **Included**  **(Y/N)** | **Definition** | **Weights** | **Percentile Rank** |
| 1 | Angelis A et al. | 2017 | UK | Metastic colorectal cancer | Not reported | Swing weighting | 9 | No | N.A | N.A | N.A | No | N.A | N.A | N.A |
| 2 | Badia X et al. | 2018 | Europe | Oncology | Patient | NPA | 11 | Yes | EVIDEM | 4.5/5 | 73% | Yes | EVIDEM | 3.3/5 | 41% |
| 3 | Badia X et al. | 2017 | Spain | Pharmaceutical drugs (general) | Clinical | NPA | 9 | Yes | EVIDEM | 4.4/5 | 72% | No | N.A | N.A | N.A |
| 4 | Baltussen R et al. | 2006 | Ghana | Sector wide | Not reported | DCE | 6 | Yes | QALY ≤5 | 0.72 | 33% | No | N.A | N.A | N.A |
| 5 | Baltussen R et al. | 2007 | Nepal | Sector wide | Not reported | DCE | 6 | Yes | QALY ≤2 | 1.08 | 50% | Yes | “Few” <100,000 | 0.97 | 17% |
| 6 | Chitama D et al. | 2011 | Tanzania | Family planning, maternal, newborn and child health interventions | Not reported | N.A | 15 | Yes | Not reported | Not weighed | N.A | Yes | Not reported | Not weighed | N.A |
| 7 | Defechereux T el al. | 2012 | Norway | Sector wide | Policy | DCE | 7 | Yes | QALY ≤2 | 1.08 | 43% | Yes | “Few” <100,000 | 0.3714 | 29% |
| 8 | Diaby V et al. | 2011 | Côte d’Ivoire | Sector wide | Policy | DCE | 4 | Yes | Disability Weights from WHO Global Burden of Disease update | 2.52 | 50% | No | N.A | N.A | N.A |
| 9 | Garau M et al. | 2018 | Italy | Pharmaceutical drugs (general) | Not reported | HPA | 13 | Yes | EVIDEM | Clinician: 0.15 | 92% | Yes | EVIDEM | Clinician:0.1 | 77% |
|  |  |  |  |  |  |  |  |  |  | Payer: 0.08 | 54% |  |  | Payer: 0.05 | 15% |
|  |  |  |  |  |  |  |  |  |  | Patient: 0.11 | 92% |  |  | Patient: 0.07 | 38% |
| 10 | Ghandour R et al. | 2014 | Palestine, Syria, Tunisia, Turkey | Cardiovascular disease | Policy | N.A | 5 | No | N.A | N.A | N.A | No | N.A | N.A | N.A |
| 11 | Gilabert-Perramon A et al. | 2017 | Catalonia | Rare disease | Not reported | HPA | 13 | Yes | EVIDEM | 0.15 | 92% | Yes | EVIDEM | 0.02 | 0% |
| 12 | Goetghebeur M et al. | 2010 | Canada | Sector wide | Societal | NPA | 15 | Yes | EVIDEM | 4.1/5 | 47% | Yes | EVIDEM | 3.1/5 | 7% |
| 13 | Golan O et al. | 2012 | Israel | Health technology | Societal | N.A | 5 | No | N.A | N.A | N.A | No | N.A | N.A | N.A |
| 14 | Guarga L et al. | 2019 | Spain | Rare disease | Not reported | NPA | 10 | Yes | EVIDEM | 4.7/5 | 90% | No | N.A | N.A | N.A |
| 15 | Inotai A et al. | 2018 | Indonesia | Pharmaceutical drugs (off patent) | Not reported | modified SMART | 6 | No | N.A | N.A | N.A | No | N.A | N.A | N.A |
| 16 | Iskrov G et al. | 2016 | Bulgaria | Rare disease | Not reported | NPA | 11 | Yes | a) Chronic life threatening  b) Acute  c) Chronic non-life threatening | 53/100 | 91% | No | N.A | N.A | N.A |
| 17 | Iskrov G et al. | 2016 | Bulgaria | Pharmaceutical drugs (general) | Not reported | Voting (Yes/No) survey | 15 | Yes | Not reported | 45/100 | 0% | Yes | Not reported | 65/100 | 7% |
| 18 | Iskrov G et al. | 2013 | Bulgaria | Sector wide | Not reported | N.A | 18 | Yes | Not reported | Not weighed | N.A | Yes | Not reported | Not weighed | N.A |
| 19 | Jankauskiene D et al. | 2013 | Lithuania | Sector wide | Not reported | NPA | 5 | No | N.A | N.A | N.A | No | N.A | N.A | N.A |
| 20 | Jaramillo H.E.C et al. | 2016 | Columbia | Sector wide | Not reported | Borda Count | 14 | Yes | EVIDEM | 0.093 | 93% | Yes | EVIDEM | 0.089 | 86% |
| 21 | Jehu-Appiah C et al. | 2008 | Ghana | Sector wide | Not reported | DCE | 5 | Yes | QALY≤5 | 0.059 | 40% | Yes | “Few” <100,000 | 0.029 | 20% |
| 22 | Jimenez A et al. | 2017 | Spain | Pulmonary arterial hypertension | Not reported | HPA | 12 | Yes | WHO Functional Classification | 0.089 | 92% | No | N.A | N.A | N.A |
| 23 | Kolasa K et al. | 2016 | Poland | Rare disease | Not reported | N.A | 10 | Yes | a) high mortality often with poor prognosis  b) chronic without high mortality and morbidity  c) severe invalidity, severely harm of capacities central to individuals’ functioning in society | Not weighed | N.A | Yes | a) prevalence < 0,5 per 10,000 UE citizens  b) prevalence in the range of 0,5 and 1 per 10,000 UE citizens  c) prevalence >1 per 10,000 UE citizens | Not weighed | N.A |
| 24 | Kroese M et al. | 2010 | UK | Genetic tests | Not reported | NPA | 5 | No | N.A. | N.A. | N.A | No | N.A. | N.A. | N.A |
| 25 | Kwon S.H et al. | 2017 | South Korea | Oncology drugs | Societal | AHP | 8 | Yes | Five year survival rate <80% | General public: 0.151 | 63% | Yes | “Rare disease” <5,000 patients | General public: 0.123 | 50% |
|  |  |  |  |  |  |  |  |  |  | Healthcare professional: 0.131 | 63% |  |  | Healthcare professional: 0.084 | 25% |
| 26 | Marsh K et al. | 2013 | UK | Preventive health intervention | Policy | DCE | 5 | No | N.A. | N.A. | N.A. | Yes | Not reported | N.A. | N.A. |
| 27 | Menon D et al. | 2008 | Canada | Health technology | Not reported | NPA | 10 | No | N.A. | N.A. | N.A. | No | N.A. | N.A. | N.A. |
| 28 | Mirelman A et al. | 2012 | Brazil, Cuba, Uganda, Norway, Nepal | Sector wide | Not reported | DCE | 7 | Yes | QALY ≤2 | All: 0.468 Brazil: 0.501; Cuba:−0.158; Uganda: 0.698; Norway: 0.682; Nepal: 0.538 | 71% | Yes | Brazil, Cuba, Uganda, Norway : “Few” <10% of population  Nepal: “few” <100K could potentially benefit | All: 0.354; Brazil: 0.660; Cuba: −1.032; Uganda: 0.355; Norway: 0.238 ; Nepal:0.48 | 29% |
| 29 | Oortwijn W.J. et al. | 2002 | Netherlands | Sector wide | Not reported | N.A | 6 | Yes | Rating scale (0–1.00), 0 represents the treatment strategies highest burden of disease and 1.00 represents the lowest burden of disease | N.A. | N.A. | Yes | Absolute numbers (per year)  a) 0–5000  b) 5001–10,000  c) 10,001–15,000  d) 15,001–20,000  e) >20,000 | N.A. | N.A. |
| 30 | Paolucci F et al. | 2015 | China | Sector wide | Policy | DCE | 7 | Yes | QALY ≤2 | 0.0384 | 0% | Yes | “Few” <100,000 | 0.301 | 86% |
| 31 | Pooripussarakul S et al. | 2016 | Thailand | Vaccines | Societal | DCE | 7 | Yes | Disease causing death, permanent disability, or long-term effects that require continuity of treatment | 2.003 | 86% | Yes | Not reported | 1.33 | 57% |
| 32 | Schey C et al. | 2017 | UK | Rare disease | Not reported | N.A | 9 | Yes | a) Morbidity  b) Mortality, severe invalidity in adulthood  c) Mortality/ severe invalidity as an infant | N.A. | N.A. | Yes | a) 1:2,000–1:20,000  b) 1:20,000 - 1:200,000  c) <1:200,000 | N.A. | N.A. |
| 33 | Sussex J et al. | 2013 | UK | Rare disease | Societal | HPA | 10 | Yes | No | N.A. | N.A. | No | N.A. | N.A. | N.A. |
| 34 | Tanios N et al. | 2013 | N.A. | Sector wide | Not reported | NPA | 43 | Yes | No | 4.2/5 | 88% | Yes | Not reported | 4.1/5 | 77% |
| 35 | Venhorst K et al. | 2014 | N.A. | Breast cancer | Not reported | HPA | 10 | No | N.A. | N.A. | N.A. | No | N.A. | N.A. | N.A. |
| 36 | Wagner M et al. | 2017 | France, Italy, Spain | Sector wide | Not reported | HPA | 12 | Yes | EVIDEM | France: 0.103 | 92% | Yes | EVIDEM | France: 0.087 | 42% |
|  |  |  |  |  |  |  |  |  |  | Italy: 0.098 | 83% |  |  | Italy: 0.083 | 50% |
|  |  |  |  |  |  |  |  |  |  | Spain: 0.089 | 42% |  |  | Spain: 0.057 | 8% |
| 37 | Wagner M et al. | 2016 | N.A. | Rare disease | Not reported | HPA | 15 | Yes | a) impact on life-expectancy  b) impact on mobility  c) impact on patient QOL  d) impact on caregiver QOL | N.A. | N.A. | Yes | EVIDEM | N.A. | N.A. |
| 38 | Youngkong S et al. | 2010 | Thailand | HIV/AIDS | Not reported | DCE | 5 | No | N.A. | N.A. | N.A. | No | N.A. | N.A. | N.A. |
| 39 | Youngkong S et al. | 2012 | Thailand | Sector wide | Not reported | N.A | 6 | Yes | Based on health state valuations, with a range from 0 (worst health status) to 1 (best health status) | N.A. | N.A. | Yes | a) ≤10,000  b) 10,001–50,000  c) 50,001–100,000  d) 100,001–500,000  e) >500,000 | N.A. | N.A. |
| 40 | Youngkong S et al. | 2012 | Thailand | HIV/AIDS | Not reported | DCE | 5 | No | N.A. | N.A. | N.A. | No | N.A. | N.A. | N.A. |

# DCE: Discrete Choice Experiment; NPA: Non Hierarchical Point allocation; HPA: Hierachical Point Allocation; AHP: Analytic Hierarchy Process; SMART: Simple Multi-Attribute Rating Technique
