# Supplementary material for: The relative importance of severity and rarity criteria in health resource allocation: an umbrella review
Source: Int J Technol Assess Health Care. 2024 Nov 14;40(1):e54. doi: 10.1017/S0266462324004653 (PMC11579674; doi:10.1017/S0266462324004653)
Supplement: Chan et al. supplementary material [file S0266462324004653sup001.zip › Supplementary file 6_CHAN.docx]

|  | **Supplementary Table 6 – Regression analysis** | | | | | | | | | | | | | | | |
| --- | --- | --- | --- | --- | --- | --- | --- | --- | --- | --- | --- | --- | --- | --- | --- | --- |
|  | Logistic Regression (N=40) | | | Linear Regression (N=25) | | | Sensitivity Analysis – severity (N=31) | | | | | Sensitivity Analysis – rarity (N=25) | | | | |
|  | Univariate analysis | | | Univariate analysis | | | Univariate Analysis | | | Multivariate Analysis | | Univariate Analysis | | | Multivariate Analysis | |
|  | Y: Severity only (N=6) | | | Y: Ratio of severity over rarity | | | Y: Weight of severity | | | Y: Weight of severity | | Y: Weight of severity | | | Y: Weight of severity | |
| Variables | N(%) | Coefficient | P value | N(%) | Coefficient | P value | N(%) | Coefficient | P value | Coefficient | P value | N(%) | Coefficient | P value | Coefficient | P value |
| **Year** |  |  |  |  |  |  |  |  |  |  |  |  |  |  |  |  |
| 2002-2014 | 14 (35.0%) | Ref |  | 11 (44.0%) | Ref |  | 13 (41.9%) | Ref |  |  |  | 11 (44.0%) | Ref |  |  |  |
| 2015-2019 | 26 (65.0%) | 0.568 | 0.544 | 14 (56.0%) | 0.352 | 0.546 | 18 (58.1%) | -0.352 | 0.204 |  |  | 14 (56.0%) | -0.209 | 0.274 |  |  |
| **Level of priority setting** |  |  |  |  |  |  |  |  |  |  |  |  |  |  |  |  |
| Supranational & national | 37 (92.5%) | Ref |  | 24 (96.0%) | Ref |  | 29 (93.5%) | Ref |  |  |  | 24 (96.0%) | Ref |  |  |  |
| Subnational | 3 (7.5%) | 1.1 | 0.404 | 1 (4.0%) | 6.152 | 0.000  # | 2 (6.5%) | -0.11 | 0.845 |  |  | 1 (4.0%) | -0.35 | 0.473 |  |  |
| **Country income** |  |  |  |  |  |  |  |  |  |  |  |  |  |  |  |  |
| High income | 22 (55%) | Ref |  | 13 (52.0%) | Ref |  | 17 (54.8%) | Ref |  |  |  | 13 (52.0%) | Ref |  |  |  |
| Others | 18 (45%) | -0.568 | 0.544 | 12 (48.0%) | -1.02 | 0.069  * | 14 (45.2%) | 0.505 | 0.0619  * | -0.0557 | 0.843 | 12 (48.0%) | 0.331 | 0.0752  * | 0.0965 | 0.534 |
| **Funding status** |  |  |  |  |  |  |  |  |  |  |  |  |  |  |  |  |
| Yes | 22 (55%) | Ref |  | 14 (56.0%) | Ref |  | 17 (54.8%) | Ref |  |  |  | 14 (56.0%) | Ref |  |  |  |
| No/not mentioned | 18 (45%) | 0.125 | 0.888 | 11 (44.0%) | -0.602 | 0.298 | 14 (45.2%) | 0.135 | 0.628 |  |  | 11 (44.0%) | -0.0295 | 0.879 |  |  |
| **Domain of prioritization** |  |  |  |  |  |  |  |  |  |  |  |  |  |  |  |  |
| Sector wide | 19 (47.5%) | Ref |  | 16 (64.0%) | Ref |  | 17 (54.8%) | Ref |  |  |  | 16 (64.0%) | Ref |  |  |  |
| Specific | 21 (52.5%) | 1.86 | 0.106 | 9 (36.0%) | 0.885 | 0.134 | 14 (45.2%) | -0.535 | 0.848 |  |  | 9 (36.0%) | -0.226 | 0.91 |  |  |
| **Perspective** |  |  |  |  |  |  |  |  |  |  |  |  |  |  |  |  |
| Policy/healthcare/payer | 8 (20.0%) | Ref |  | 5 (20.0%) | Ref |  | 7 (22.6%) | Ref |  |  |  | 5 (20.0%) | Ref |  |  |  |
| Societal/public/patient | 6 (15.0%) | -0.693 | 0.615 | 5 (20.0%) | 0.06 | 0.949 | 6 (19.4%) | 0.247 | 0.561 |  |  | 5 (20.0%) | 0.378 | 0.216 |  |  |
| Not mentioned | 26 (65.0%) | -1.076 | 0.3 | 15 (60.0%) | 0.086 | 0.911 | 18 (45.0%) | -0.218 | -0.65 |  |  | 15 (60.0%) | 0.167 | 0.498 |  |  |
| **Objective** |  |  |  |  |  |  |  |  |  |  |  |  |  |  |  |  |
| Rank ordering/ Reimbursement decision making | 32 (80.0%) | Ref |  | 18 (72.0%) | Ref |  | 23 (74.2%) | Ref |  |  |  | 18 (72.0%) | Ref |  |  |  |
| Normative study | 8 (20.0%) | -0.336 | 0.775 | 7 (28.0%) | -0.57 | 0.374 | 8 (25.8%) | -0.261 | 0.407 |  |  | 7 (28.0%) | -0.163 | 0.445 |  |  |
| **Source of criteria** |  |  |  |  |  |  |  |  |  |  |  |  |  |  |  |  |
| Proposed | 15 (37.5%) | Ref |  | 6 (24.0%) | ref |  | 8 (25.8%) | Ref |  |  |  | 6 (24.0%) | Ref |  |  |  |
| Adapted from existing study | 25 (62.5%) | 0.0465 | 0.961 | 19 (76.0%) | 0.268 | 0.693 | 23 (74.2%) | -1.01 | 0.0004  *** | -0.877 | 0.002  *** | 19 (76.0%) | -0.734 | 0.000  *** | -0.688 | 0.001  *** |
| **Preference elicitation technique** |  |  |  |  |  |  |  |  |  |  |  |  |  |  |  |  |
| DCE | 13 (32.5%) | Ref |  | 11 (44.0%) | ref |  | 12 (38.7%) | Ref |  |  |  | 11 (44.0%) | Ref |  |  |  |
| Others | 27 (67.5%) | 0.963 | 0.405 | 14 (56.0%) | 0.279 | 0.632 | 19 (61.3%) | -0.675 | 0.0124  ** | -0.482 | 0.094  * | 14 (56.0%) | -0.238 | 0.2103 |  |  |
| **Participants in criteria setting** |  |  |  |  |  |  |  |  |  |  |  |  |  |  |  |  |
| Heterogeneous | 14 (35.0%) | Ref |  | 8 (32.0%) | ref |  | 11 (35.5%) | Ref |  |  |  | 8 (32.0%) | Ref |  |  |  |
| Homogeneous | 13 (32.5%) | -0.405 | 0.69 | 7 (28%) | -0.67 | 0.378 | 9 (29.0%) | -0.277 | 0.392 |  |  | 7 (28%) | -0.249 | 0.292 |  |  |
| Not applicable (set of criteria cited from other studies) | 13 (32.5%) | -1.28 | 0.298 | 10 (40.0%) | -0.512 | 0.461 | 11 (35.5%) | -0.728 | 0.023  ^ |  |  | 10 (40.0%) | -0.43 | 0.054  ^ |  |  |
| **Participants in criteria weighing** |  |  |  |  |  |  |  |  |  |  |  |  |  |  |  |  |
| Heterogeneous | 15 (37.5%) | Ref |  | 10 (40.0%) | ref |  | 13 (41.9%) | Ref |  |  |  | 10 (40.0%) | Ref |  |  |  |
| Homogeneous | 19 (47.5%) | -0.31 | 0.733 | 15 (60.0%) | -0.386 | 0.513 | 18 (58.1%) | 0.00696 | 0.98 |  |  | 15 (60.0%) | -0.229 | 0.2358 |  |  |
| Not applicable (no weighing) | 6 (15.0%) | -15.81 | 0.994 | N.A. | N.A. | N.A. | N.A. | N.A. | N.A. |  |  | N.A. | N.A. | N.A. |  |  |

*P-value <0.1

**P-value <0.05

***P-value <0.01

# ‘Level of setting’ not added to the final model due small number of observation in ‘subnational’ category despite fulfilling the criteria in forward stepwise process

^ ‘Participants in criteria setting’ not added to the final model as relationship appears to be driven by the ‘not applicable’ category.
